# Chronic Intraventricular Cannulation for the Study of Glymphatic Transport

**DOI:** 10.1523/ENEURO.0537-24.2025

**Published:** 2025-06-17

**Authors:** Daniel Gahn-Martinez, Michael Giannetto, Ethan Chang, Nathaniel Beam, Paul Tobin, Virginia Plá, Maiken Nedergaard

**Affiliations:** ^1^Center for Translational Neuromedicine, University of Copenhagen, Copenhagen 2200, Denmark; ^2^Center for Translational Neuromedicine, University of Rochester Medical Center, Rochester, New York 14642

**Keywords:** clearance, CSF, glymphatic flow, glymphatic system, intraventricular cannulation, tracer delivery

## Abstract

Glymphatic transport in rodents has primarily been studied using cisterna magna cannulation (CMC), a minimally invasive method for cerebrospinal fluid (CSF) tracers infusion. However, CMC is suboptimal due to the lack of bony structures to stabilize the cannula, leading to potential movement artifacts. Here, we present an alternative approach involving chronic cannulation of the lateral ventricles of mice for CSF tracer delivery. A direct comparison demonstrated that intraventricular cannulation (IVC) reproduces CMC results in vivo, including perivascular labeling of the middle cerebral artery, which was further confirmed by ex vivo analysis. IVC enables tracer infusion in awake mice, facilitating glymphatic transport studies in conjunction with behavioral assessments that were previously unattainable. Additionally, IVC supports repeated infusions in awake animals, offering the potential to reduce the number of experimental animals required. This study establishes IVC as a robust alternative for studying glymphatic transport and associated physiological processes.

## Graphical abstract

**Figure:**
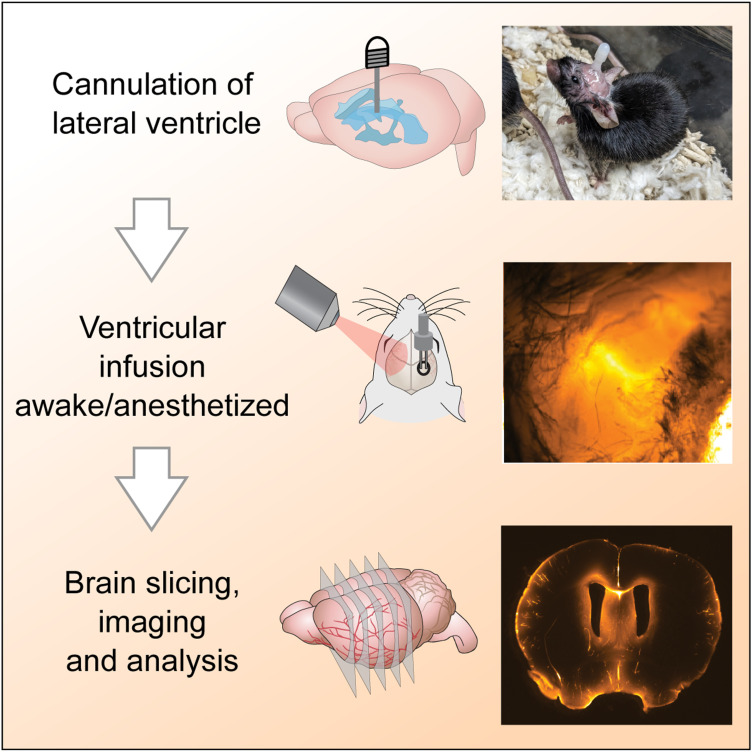
Visual Abstract

## Significance Statement

Refinement in tracer delivery methods is crucial for advancing the study of brain fluid transport, a field traditionally reliant on these techniques to visualize cerebrospinal fluid movement. Cisterna magna cannulation has become the gold standard for assessing glymphatic system function due to its minimal invasive nature and high reproducibility. In this study, we present an alternative method for tracer delivery to the lateral ventricles through the placement of chronically implanted cannulas. Additionally, we demonstrate that intraventricular cannulation is well suited for longitudinal evaluation of glymphatic system function and can potentially be integrated with cognitive or motor skill testing.

## Introduction

Cerebrospinal fluid (CSF) is primarily produced by the choroid plexuses, located in the lateral ventricles as well as in the third and fourth ventricles (Rasmussen et al., 2022). From these sites, CSF flows into the cisterna magna (CM), a CSF-filled space at the base of the cerebellum, and circulates to the subarachnoid space. CSF then enters the brain via the perivascular spaces (PVSs) of the subarachnoid arteries and penetrating arterioles, where it disperses into the parenchyma, facilitated by aquaporin-4 (AQP4) water channels, and mixes with the interstitial fluid (ISF; Mestre et al., 2017). Waste-laden CSF subsequently exits the brain via the PVS surrounding veins, meningeal lymphatic vessels, cranial and spinal nerves, and ultimately returns to the circulatory system (Benveniste et al., 2017). Dysfunction of the glymphatic system, which relies on this polarized flow path to clear brain solutes, has been implicated in several neurodegenerative diseases, including Alzheimer's disease (Peng et al., 2016; Beschorner and Nedergaard, 2024). Furthermore, excessive CSF inflow or impaired outflow contributes to edema in conditions such as ischemic stroke and traumatic brain injury (Mestre et al., 2020). Quantifying glymphatic fluid transport could provide critical insight and foster the development of novel treatment for neurological diseases.

The study of CSF circulation commonly relies on the use of a tracer—radioactive, magnetic, or fluorescent—to visualize flow dynamics. Cisterna magna cannulation (CMC) has emerged as a preferred method due to its minimal interference with normal brain function and ease of access. CMC is widely used to visualize glymphatic function through in vivo imaging techniques (e.g., 2-photon or macroscopic imaging) or by preparation of vibratome sections after brain harvest (Xavier et al., 2018). Yet, even with successful chronic cannula implantation protocols, CMC has limitations: it restricts the normal head movement in mice and carries a risk of damaging the cerebellum or brain stem.

These drawbacks highlight the need for improved methodologies, particularly for studies combining glymphatic analysis with behavioral assays in freely moving mice. Importantly, the glymphatic system is highly sensitive to invasive procedures, with acute cannula implantation causing a significant reduction in its function (Mestre et al., 2018). This underscores the necessity of a technique that allow the recovery of baseline CSF circulation postsurgery (Plá et al., 2022).

As an alternative to CMC, we explored intraventricular cannulation for glymphatic studies. This approach offers several advantages, including secure attachment, enabling free movement of mice after surgery. Furthermore, intraventricular cannulation supports repeated tracer injections, paving the way for new avenues of longitudinal glymphatic research.

## Materials and Methods

### Presurgery cannula and solutions preparation

To minimize the duration for the surgical procedure, it is advisable to prepare the lines, cannula implants, and appropriate tracer dilutions in advance. Tracers, such as fluorochrome-associated dextran or bovine serum albumin (BSA) are commonly used at a concentration of 0.5% (w/v) in artificial CSF as a solvent. These preparations will be described as examples in the protocol.

### Prepare lines and cannula implants

**Figure 1.**
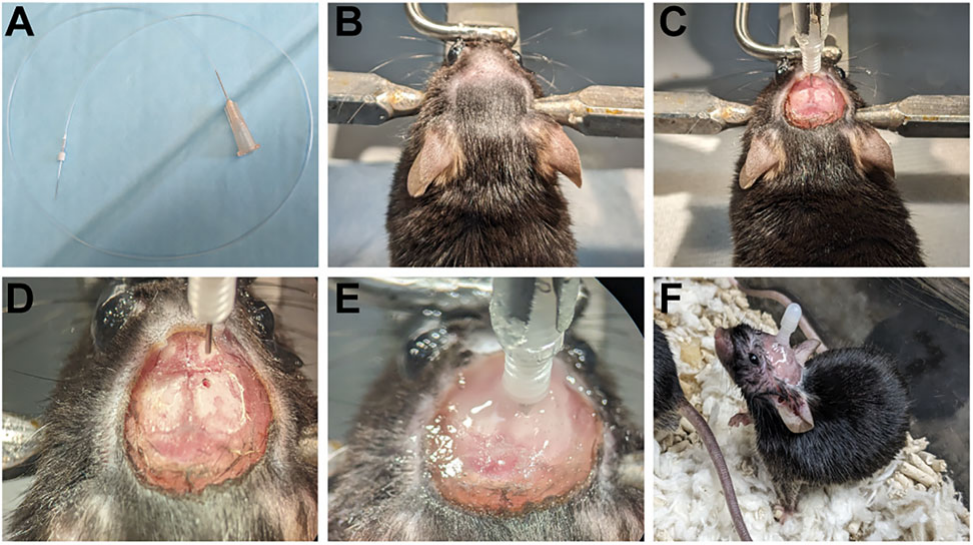
Chronic intraventricular cannula placement. ***A***, 30G needle inserted into PE10 tubing attached to 26G internal cannula. ***B***, The anesthetized mouse is fixed in the stereotaxic frame. The head is leveled using the bite bar holding the nose. ***C***, The dorsal side of the skull is exposed. Bregma and lambda are visible and leveled. ***D***, Starting from Bregma, the coordinates AP −0.6 mm, ML 1.2 mm are located and marked. A hole is drilled in the skull, exposing the brain cortex. ***E***, The cannula is lowered DV −2.0 mm and fixed in place using a mix of glue and dental cement. ***F***, The mouse fully recovers after surgery.

1. Carefully, introduce ∼5 mm of a ∼40-cm-long piece of PE10 tubing (Polyethylene 0.011′′ × 0.024′′ tubing, Braintree Scientific, PE10) into a ∼1-cm-long piece of PE50 tubing (BD Intramedic PE Tubing-PE50, Fisher Scientific, 14-170-12B).
  NOTE: If planning to do awake infusions, the length of the PE10 tubing should be longer enough to compensate for the wide range of movement that the mouse will require.
2. Introduce the beveled end of a 26G internal cannula into the other end of the PE50 tubing and further insert the cannula into the PE10 tubing. NOTE: The PE50 tubing acts as an outer connector between the PE10 tubing and the 26G internal cannula, preventing leaks during the infusion and reinforcing the union between both parts
3. Introduce a 30G needle (Fisher Scientific, 14826F) at the other end of the PE10 tubing, being careful not to puncture the tubing at any point (Fig. 1*A*). Optionally, the sharp edge can be removed by breaking the needle with forceps to avoid puncture risk.
4. Set aside an equal number of dummy cannulas with 0.1 mm projection (Plastics One, C315DC/SP) tightly screwed into a 26G guide cannula (Plastics One, C315G SP). NOTE: Failing to secure the dummy and guide cannula could result in the dummy falling out during the recovery period in which the animals will behave freely in their home cage.

### Prepare aliquots of BSA-647 tracer in artificial cerebrospinal fluid

1. Add 500 µl of artificial cerebrospinal fluid (aCSF; in mM: 26 NaCl, 2.5 KCl, 1.25 NaH_2_PO_4_, 2 MgSO_4_, 2 CaCl_2_, 10 glucose, 26 NaHCO_3_, pH 7.4) to a 5 mg bottle of bovine serum albumin, Alexa Fluor 647 conjugate (BSA-647; Fisher Scientific, A34785) to make a 1% wt/vol solution.
2. Prepare 20 µl of aliquots and store the tubes at −80°C.

### Prepare Ketamine/Xylazine anesthesia

Circadian rhythms have been shown to affect the glymphatic system (Hablitz et al., 2020). To compensate for the possible fluctuations, all experiments were performed during the daylight, matching the resting period of the mice and thus, the periods of higher glymphatic activity. For the same reason, a mixture of Ketamine/Xylazine was chosen as anesthetics, since its application closely replicates the natural flow of glymphatic movement in naturally sleeping mice (Hablitz et al., 2020).

1. Use 1 ml of 100 mg/ml Ketamine HCl (UR Pharmacy, NDC 0409-2051-05), 0.25 ml of 20 mg/ml Xylazine (AnaSed Injection), and 5 ml of phosphate-buffered saline BioPerformance Certified, pH 7.4 (PBS; Sigma Aldrich, P5368-5X10PAK) to make a solution of Ketamine–Xylazine (KX).
2. Store at 4°C for up to 7 d.

### Prepare carprofen analgesia

1. Use 0.1 ml of carprofen (Rimadyl Sterile Injectable Solution, 50 mg/ml) and 4.9 ml of PBS to make a solution of carprofen.
2. Store at 4°C for up to 7 d.

#### Cannula implant surgery

Implantation protocol can be optimized to a maximum duration of 20 min per animal, counting with the anesthesia induction period, which results in a very efficient procedure. Different target areas can be used, including parenchyma as shown in Plá et al. (2022).

1. During the whole procedure, follow your animal care unit directions to ensure deep anesthesia is reached and that pain is minimized during and after the surgery and to ensure full recovery. Remember to use proper sterile techniques to avoid any surgical complications and ensure animal well-being.
  - Weigh and anesthetize a mouse of either sex (C57BL6, 8 weeks old, Charles River Laboratories) with KX (Ketamine 100 mg/kg, Xylazine 20 mg/kg), 10 µl/g via intraperitoneal injection (i.p.). Check the depth of anesthesia with a toe pinch. If necessary, the mouse should be redosed subdermally with 100 µl of KX.
  - Alternatively, anesthetize using an isoflurane induction chamber at 3% isoflurane, ∼1 L/min O_2_. Once the mouse is asleep, it should be rapidly transported to the surgery station, where a nose cone maintains a flow of isoflurane anesthesia at 1.5–2% for the rest of the surgery.
2. Once the surgical plane is reached, use a trimmer to shave the head of the mouse. The dorsal surface, between the ears and the eyes, should be completely shaved.
3. Place the mouse on a stereotaxic frame. Use ear bars to gently secure the mouse from both sides of the head and use a nose bar to make sure that the head is leveled (Fig. 1*B*).
  NOTE: This is a crucial step of the surgery. If the head is not leveled, the cannula may not be implanted in the ventricle.
4. Once the head is secured, sterilize the area using alcohol, moving in circles, from the center of the head outwards. Repeat the process using iodine and finalize by repeating with alcohol again.
5. Apply eye ointment (Puralube, Fisher Scientific) to both eyes to keep them moisturized during the surgery.
6. Give the mouse adequate presurgery analgesia [e.g., carprofen (5 mg/kg) subdermal 5 µl/g].
7. Using a dissection microscope, use forceps (Fine Science Tools, 91197-00) to lift a small portion of the skin between the ears, and use fine scissors (Fine Science Tools, 91460-11) to cut the skin and expose the dorsal section of the skull (Fig. 1*C*). Ideally, both bregma and lambda should be visible. If the mouse will be used for in vivo imaging, only remove skin covering half skull that will have the implant.
8. Place a cannula holder (Stoelting, 51636) into the stereotaxic arm, and carefully insert the previously prepared 26GA cannula guide with dummy screwed in into the holder. Make sure the cannula is secured to the arm by tightening one of the screws on the side of the holder.
9. Use stereotaxic technique to locate the implantation site, using bregma as a reference.
  - By using the XYZ control of the stereotaxic, find and place the tip of the cannula on top of the bregma. Record the coordinates on the stereotaxic arm.
  - Move the tip of the cannula to lambda and record all stereotaxic coordinates (anterior/posterior, AP; medial/lateral, ML; dorsal/ventral, DV).
  - Compare lambda coordinates to the bregma ones. If the skull is leveled, there will not be >0.1 mm difference in the DV axis between both. Otherwise, adjust the angle of the mouse head by moving the bite bar holding the nose or readjust the ear bars accordingly.
  - Finally, move the cannula to the coordinates AP −0.6 mm, ML −1.2 mm relative to Bregma (for lateral ventricle implant), and mark the position by scoring the skull surface with fine forceps.
10. Drill a burr hole:
  - Using a drill (Ram Products, 106ADIG) and a 0.5 mm microdrill burr, drill a hole at the desired spot until the brain tissue is visible (Fig. 1*D*). Optionally, a dust cleaner spray can be used to eliminate any bone particles.
  - Ensure the dura is broken by inserting a 30 G needle tip at a steep angle into the burr hole, and gently lifting around the hole. This will also help remove any remaining pieces of skull from the drilling. Ideally, little bleeding results from this procedure. Make note if there is excessive bleeding that is not immediately resolved by blotting with a Kimwipes or cotton swab. Any bleeding must be resolved before proceeding with the next step.
11. Carefully lower the cannula into the skull, stopping once it reaches the same level as the brain surface. Once there, note the DV coordinates on the stereotaxic arm, and lower the cannula to DV −2.0 mm.
12. Using a mix of glue and dental cement, secure the cannula in place. The surrounding exposed area of the skull should also be covered in a layer of the mixture to protect the skull from the environment (Fig. 1*E*). NOTE: Be careful not to glue the dummy cannula to the guide cannula, the guide cannula will need to be unscrewed later to insert the internal cannula.
13. To ensure a full recovery, the following steps are recommended:
  - Place the mouse in a clean cage. Optionally, part of their original cage bedding can be added for comfort. A 37°C circulating warm water pad should be placed under the cage (Gaymar, TP700) and should be placed at the bottom of the cage to work as a heating source. Optionally this can be maintained on the first night postsurgery to help mice to regulate their temperature, especially if the animal is in weak condition (e.g., aged mice). Close monitor the animal until it recovers from anesthesia (Fig. 1*F*).
  - To avoid bumping the cannula and the consequent possible damage to the brain, remove the metal bars containing food and water typically at the top of the cage, or any non-compatible enrichment housing or bedding. Optionally, use Nutra-Gel Complete Nutrition as a food and water source. If used, Nutra-Gel should be provided daily for the duration of the mouse recovery.
  - Provide adequate analgesia for the entire recovery period. For example, carprofen (5 mg/kg) should also be administered subdermally every 24 h for the first 72 h postsurgery.

NOTE: Anesthesia, analgesia, and recommendations may vary depending on each institution's animal care and use committee.

#### Tracer infusion, in vivo imaging, and brain extraction

##### Load tracer solution into line

1. Fill a Hamilton 100 µl gastight syringe (for 10 µl infusion volume), or a Hamilton 10 µl (for 1 µl infusion volume) Model 1701 LT Syringe with aCSF.
  NOTE: The syringes and lines should be filled with aCSF to ensure accurate infusion volumes.
2. Connect the 30 G needle of the previously prepared line to the syringe and flush the line until all the air is pushed out of the line.
3. Introduce a small air bubble (1 cm) in the line by withdrawing slightly. This will ensure that the aCSF within the line does not mix with the tracer solution.
4. Place the tip of the cannula into the tracer tube and withdraw the desired volume to infuse. 1 or 10 µl of BSA-647 solutions have been used in these experiments.

##### Ventricular infusion

NOTE: This technique allows tracers to be delivered once (single infusions) or across several days (multiple infusions). The key difference between these deliveries is in step 5 of this section.

1. Anesthetize the mouse using either Ketamine/Xylazine or isoflurane anesthesia, same dosing as described in the Cannula implant surgery section.
2. Place your Hamilton syringe previously attached to the line into an infusion pump (Standard Infuse/Withdraw Pump 11 Elite, Harvard Apparatus, 704505 has been used as an example in the current protocol) and select the desired parameters.
  - If infusing 1 µl, use a Hamilton 10 µl Model 1701 LT Syringe, and select Infuse Only, Size: 10 µl–0.461 mm, Target: 1.0 µl, Rate: 0.2 µl/min.
  - If infusing 10 µl, use a Hamilton 100 µl gastight syringe, and select Infuse Only, Size: 100 µl–1.457 mm, Target: 10 µl, Rate: 2 µl/min.
3. Insert the infusion cannula into the mouse implanted guide cannula.
  - Grip the guide cannula with a hemostat (Fine Science Tools, 91308-12), and carefully unscrew and remove the dummy cannula.
    NOTE: Firmly grasp the guide cannula with the hemostat, this will ensure that no force from the withdrawal of the dummy or insertion of the internal cannula is transmitted to the brain.
  - Insert the internal cannula immediately, making sure that they get completely attached. There is a tactile clicking as the internal cannula seats into the guide cannula.
  - If infusing while the mouse is awake, make sure the mouse is restrained to avoid sudden movements that could affect the infusion. Take out the dummy cannula and quickly insert the internal cannula. Optionally, a drop of glue in the junction of the internal cannula and the guide cannula can be used to strengthen the union.
    NOTE: If the mice are awake during the infusion, they must be monitored all the time in case they start to chew on the line.
4. Start the infusion, recording the time and checking continuously the mouse state and anesthesia level.
5. After 30 min from the moment the pump started:
  - If you are doing single infusions: Use a hemostat to clamp the line ∼5 cm from the head of the mouse, and safely cauterize (Fisher Scientific, NC1137309) the tube proximal to the pump.
  - If you are doing multiple infusions: Follow the steps on part a to cauterize the line and use a hemostat to pull the internal cannula from the guide cannula. Quickly put the dummy back in the guide cannula, making sure that it is tightly secured.
6. If only performing glymphatic analysis, mice can be killed for brain harvest.
7. During brain dissection, use a hemostat (Fine Science Tools, 91308-12) to grip the guide cannula and pull the cannula out from the brain. In a normal procedure, both the internal and guide cannula are taken off, still attached to the glue from the skull surface.
  NOTE: Make sure to pull the cannula straight up to avoid damage to the brain tissue.
8. If glymphatic analysis is performed, brain has to be removed from the skull within 3 min from death, to avoid postmortem edema that can artifactually rise the level of penetrance of the tracers into the brain tissue (Du et al., 2021).
9. Drop-fix the brain with 4% paraformaldehyde (PFA) w/v in PBS solution. Let the brain fix overnight at 4°C.

##### In vivo imaging

1. Follow steps 1–3 from the Ventricular infusion section to anesthetize the mouse, prepare the syringe, select desired parameters, and insert the infusion cannula.
2. Cut the skin contralaterally to the implantation site, exposing the surface of the parietal bone where the middle cerebral artery is located.
3. Place the mouse under a fluorescent microscope (Olympus MVX10) and focus on the surface of the skull. NOTE: Green channel may be used to facilitate focusing of the microscope by the autofluorescence of the tissue, but the infusion data must be collected and analyzed at the adequate wavelength (e.g., Far-red channel for BSA-647).
4. Start the infusion, recording the time and checking continuously the mouse state and anesthesia level. Take a picture when the infusion starts, and once every 15 min, for up to 2 h. Redose the mouse if the mouse shows any reflexes.
5. After the imaging is done, proceed to harvest the tissue as described at the Ventricular infusion section.

#### Brain sectioning and imaging

1. In a PFA-safe hood, switch brains to PBS.
2. Carefully place the brain on a glass slide and under a fluorescent microscope (Olympus MVX10) and capture the dorsal and ventral side of the brain.
3. Removing the cerebellum with a razor blade, cut the cerebral cortex into coronal sections. Take the cerebral cortex and glue the proximal site of the cut into a vibrating blade microtome (Leica VT1200 S Fully automated, Leica, VT1200S) plate.
4. Use a speed of 1.00 mm/s, amplitude of 1.30 mm, and step size of 100 µm to slice the brain. Collect the first slice at which the corpus callosum appears connected (Bregma 1.20 mm) and every other sixth slice to get an overview of the whole brain and mount it into microscope slides (e.g., Fisherbrand Superfrost Plus, Fisher Scientific, 12-550-15).
5. Place the slides in a cool, dark space until they dry and mount them using mounting media (e.g., ProLong Gold Antifade Reagent with DAPI, Invitrogen, P36931) and cover glass (e.g., Corning Square and Rectangular Cover Glasses, Fisher Scientific, 12-553-468).
6. After the mounting media has settled, image all the slices (Olympus MVX10). Capture the adequate channel for the tracer used (e.g., Far-red for BSA-647) and, additionally, the green channel for autofluorescence, for later analysis.

#### Image analysis with ImageJ

##### Fluorescent tracer influx measured by Mean Pixel Intensity

1. Start Image J and import the .tiff images of the slices (File > Open…)
2. Using the Polygon tool, draw a region of interest (ROI) around the whole slice (Fig. 5*Ai*), making sure to avoid as much background as possible. The tool Magic Wand can be used as an alternative to the Polygon tool, but it may not always be accurate.
  NOTE: Failing to exclude background from the ROI could result in lower values in the next steps.
3. Record the ROI in the ROI Manager (Analyze > Tools > ROI Manager). It is recommended to rename each ROI based on the mouse ID and slice # for later analysis. After all the ROIs are recorded, select them, and use More > Save in the ROI manager to save them.
4. Use Analyze > Set Measurements to select the desired measurements. For the purpose of analyzing the Mean Pixel Intensity (MPI), the option Mean Grey Value must be selected.
5. Select the Slice Image and its corresponding ROI and use the option Measure of the ROI Manager to get the MPI of the slice. Copy and paste the given value into an Excel file or preferred software for later analysis. Repeat for all the brain slices.

##### Exclusion of periventricular fluorescent signal

NOTE: This step is optional and is performed to compare influx from the IVC and CMC infusions. It is not necessary in a standard IVC data analysis.

1. Start Image J and import the .tiff images of the slices (File > Open…)
2. Using the Polygon tool, draw a region of interest (ROI) around the ventricles (Fig. 5*Aiii*). Use Delete to clear the internal area of the ROI.
3. Still using the Polygon tool draw an ROI around the whole slice (Fig. 5*Ai*) and record it into the ROI Manager (Analyze > Tools > ROI Manager). It is recommended to rename each ROI based on the mouse ID and slice # for later analysis. After all the ROIs are recorded, select them, and use More > Save in the ROI manager to save them.
  NOTE: Previous ROIs from Fluorescent tracer influx measured by MPI could be used instead of drawing new ones.
4. Use Analyze > Set Measurements to select the desired measurements. For the purpose of analyzing the MPI, the option Mean Grey Value must be selected.
5. Select the Whole Slice Image and its corresponding ROI and use the option Measure of the ROI Manager to get the MPI of the slice excluding the periventricular fluorescent signal. Copy and paste the given value into an Excel file or preferred software for later analysis. Repeat for all the brain slices.

##### Ventricle size

1. Start Image J and import the .tiff images of the slices (File > Open…)
2. Using the Polygon tool, draw a region of interest (ROI) around the ventricles (Fig. 5*Aii*). The tool Magic Wand can be used as an alternative to the Polygon tool, but it may not be always accurate.
3. Record the ROI in the ROI Manager (Analyze > Tools > ROI Manager). It is recommended to rename each ROI based on the mouse ID and slice # for later analysis. After all the ROIs are recorded, select them, and use More > Save in the ROI manager to save them.
4. Use Analyze > Set Measurements to select the desired measurements. For the purpose of analyzing the Ventricle Size, the option Area must be selected.
5. Select the Slice Image and its correspondent ROI and use the option Measure of the ROI Manager to get the MPI of the slice. Copy and paste the given value into an Excel file or the preferred text software for later analysis. Repeat for all the brain slices.

##### In vivo tracer influx measured by Mean Pixel Intensity

1. Start ImageJ and import the .tiff images of the slices (File > Open …)
2. Using the Polygon tool, draw a region of interest (ROI) on the proximal region to the middle cerebral artery (MCA).
  NOTE: In the first few minutes after the pump starts, the tracer has not reached the MCA, which could lead to the location of the artery being hard to find. Refer to later pictures to localize the area accurately.
3. Record the ROI in the ROI Manager (Analyze > Tools > ROI Manager).
4. Use the option Measure of the ROI Manager to get the MPI of the MCA. Copy and paste the given value into an Excel file or the preferred text software for later analysis.
5. Apply the recorded ROI to the next images (in a chronological way) to measure MPI changes over time.
  NOTE: The efficiency of the ROI could be affected by the movements of the mouse during imaging. Always check that the ROI is outlining the MCA and redraw if needed.

## Results

### Intraventricular cannulation is an access point to cerebrospinal fluid

To assess whether intraventricular cannulation can effectively access CSF circulation and, by extension, the glymphatic system, cannulas were implanted into the right ventricle of mice. After a 72 h recovery period to ensure full recovery from surgery (Fig. 1), the animals were anesthetized, and the ventricles were infused with a CSF tracer, BSA-647 (66 kDa, 0.5% wt/vol in aCSF, 10 µl at 2 µl/min infusion rate).

**Figure 2.**
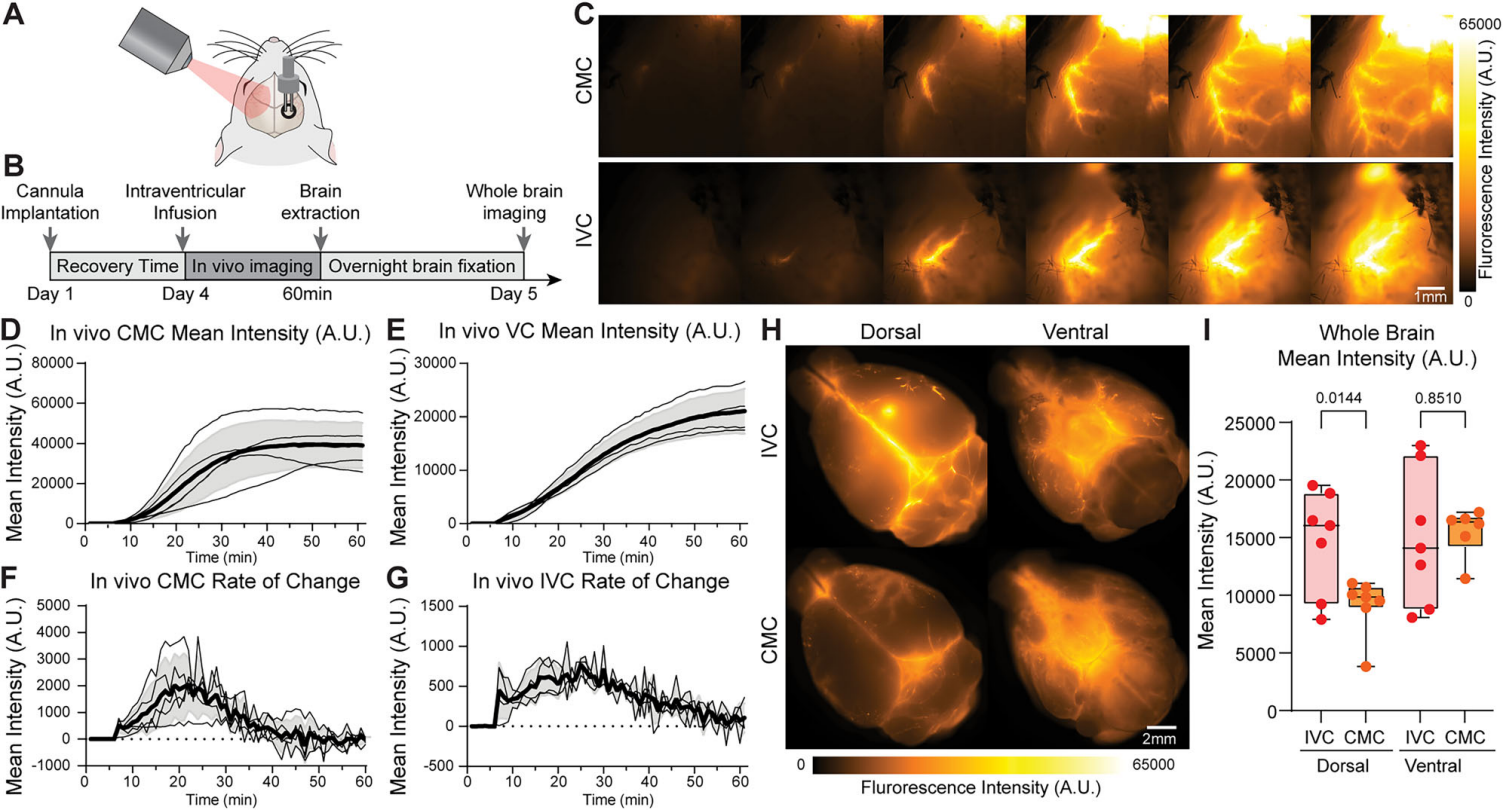
Intraventricular tracer infusion labels glymphatic flow of cerebrospinal fluid similar to cisterna magna infusion. ***A***, Diagram of the mouse head during in vivo imaging. ***B***, Timeline of the experiment. ***C***, Top, Cisterna magna (CMC) tracer infusion of bovine serum albumin conjugated to Alexa Fluor 647 (BSA-647) and in vivo imaging of dorsal middle cerebral artery (MCA). Bottom, Contralateral in vivo imaging of MCA with an IVC BSA-647 tracer infusion. IVC injected BSA-647 circulates along the brain surface and perivascular spaces of the MCA. ***D***, CMC-infused BSA-647 fluorescence around the MCA increases over time (*n* = 5). ***E***, IVC CSF tracer fluorescence around the MCA increases over time (*n* = 4). ***F***, CMC-injected BSA-647 tracer fluorescence rate of change. ***G***, IVC injected BSA-647 tracer fluorescence rate of change. ***H***, Top half, Dorsal (left) and ventral (right) BSA-647 influx in mice with 10 µl IVC infusion after 30 min of circulation time. Bottom half, Dorsal (left) and ventral (right) BSA-647 distribution on mice with 10 µl CMC Infusion after 30 min of circulation time. Scale bar, 2 mm. ***I***, Mean fluorescent intensity (A.U.) of the dorsal (*p* = 0.0144) and ventral (*p* = 0.8510) areas of the IVC- and CMC-injected brains (*n* = 4 IVC, 5 CMC). Groups were compared using unpaired two-tailed *t*-test. Statistical comparisons are included in the bar plots.

**Figure 3.**
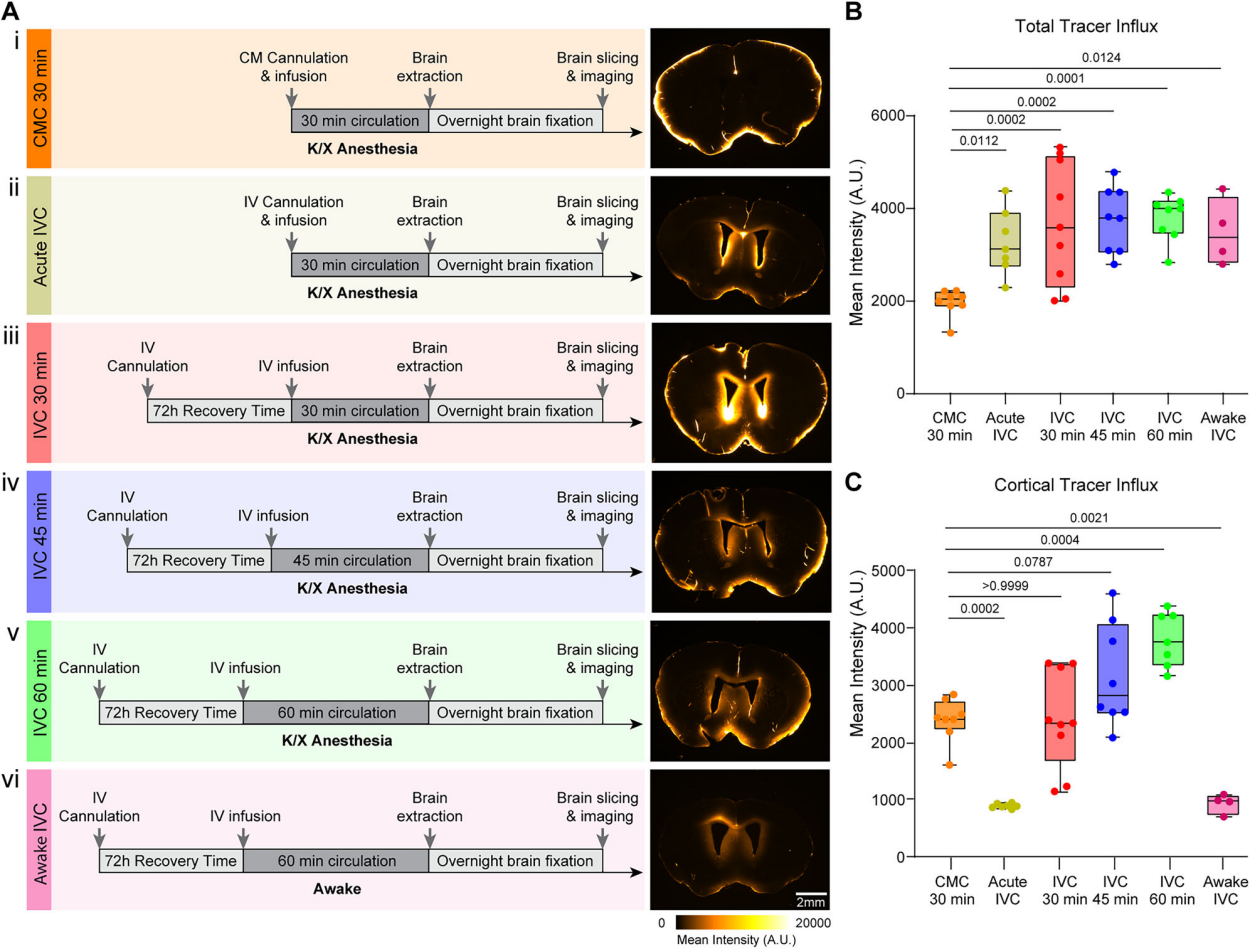
Adequate surgery recovery time is required for normal glymphatic flow. ***A***, Timelines and representative brain sections for different CSF tracer infusion paradigms. ***i***, Cisterna magna (CMC) with 30 min of circulation time, BSA-647, 10 µl, 2 µl/min. ***ii***, Acute intraventricular (IVC) with 30 min of circulation time, BSA647, 10 µl, 2 µl/min. ***iii***, IVC with 30 min of circulation time, BSA-647, 10 µl, 2 µl/min. ***iv***, IVC with 45 min of circulation time, BSA-647, 10 µl, 2 µl/min. ***v***, IVC with 60 min of circulation time, BSA-647, 10 µl, 2 µl/min. ***vi***, Awake IVC with 60 min of circulation time, BSA-647. ***B***, Mean fluorescent intensity (A.U.) of BSA-647 in the entire brain sections from each injection paradigm. ***C***, Mean fluorescent intensity of cortical BSA-647 from each injection paradigm. CMC, 30 min of circulation time (*n* = 8); acute IVC, 30 min of circulation time (*n* = 7); IVC, 30 min of circulation time (*n* = 9); IVC, 45 min of circulation time (*n* = 8); IVC 10 µl, 60 min of circulation time (*n* = 8); awake IVC, 60 min of circulation time (*n* = 4). Groups were compared using ordinary one-way ANOVA. Statistical comparisons are included in the bar plots.

In vivo macroscopic imaging of the contralateral exposed mouse skull (Fig. 2*A*,*B*) showed that the CSF tracer traveled along the perivascular space surrounding the middle cerebral artery (Fig. 2*C*), consistent with physiological glymphatic influx as previously described (Hablitz et al., 2019). The mean fluorescence intensity (A.U.) and tracer distribution along the middle cerebral artery increased over time, closely mirroring results obtained with CMC (Fig. 2*D–G*), which was used as a reference. Furthermore, analysis of the harvested intact mouse brains showed tracer labeling of perivascular space surrounding the middle cerebral artery, its branches, and the circle of Willis (Fig. 2*H*,*I*). In fixed brain sections, the CSF tracer was observed to disperse from the periventricular tissue at the brain base and into the cortex penetrating the parenchyma (Fig. 3*Aiii*).

### Intraventricular cannulation as an alternative to cisterna magna cannulation

A significant limitation of CMC is the difficulties in securely implanting and stabilizing a chronic cannula due to the absence of bony structures near the atlantooccipital membrane. In CMC, the cannula is glued to the occipital bone at the back of the skull, leaving it vulnerable to movement-related displacement or restricting the mouse range of motion. In contrast, an IVC is securely anchored in a 360° ring around the craniotomy by dental cement/glue, providing superior stabilization. The design is ideal for CSF tracer injections in non-anesthetized mice. Yet, opening the cranial cavity can temporally impair glymphatic system function (Plá et al., 2022). To determine whether IVC yields comparable results to CMC, we conducted a formal comparison of the two methods and analyzed the outcome (Figs. 2, 3).

Tracer distribution analysis from brain slices showed that, while both methodologies exhibited overall comparable tracer distribution patterns, the directional flow of CSF prevents CMC from labeling the ventricles (Fig. 3*Ai*). In contrast, IVC infusions label the ventricles (Fig. 3*Aiii*–*v*). Acute IVC did not result in tracer labeling of the cortex (Fig. 3*Aii*), confirming that the glymphatic system is transiently impaired immediately following cannula implantation, as previously reported (Mestre et al., 2018). Similarly, awake infusions did not show cortical labeling (Fig. 3*Avi*), further supporting the established notion that the glymphatic system is less active during wakefulness (Xie et al., 2013).

When comparing total tracer distribution across the brain, IVC showed higher levels of tracer influx than CMC (Fig. 3*B*). However, when the local increased signal around the ventricles was excluded from the analysis, the patterns and levels of tracer influx were similar patterns between IVC and CMC (Fig. 3*C*).

### Intraventricular cannulations allow longitudinal assessment of the glymphatic function

**Figure 4.**
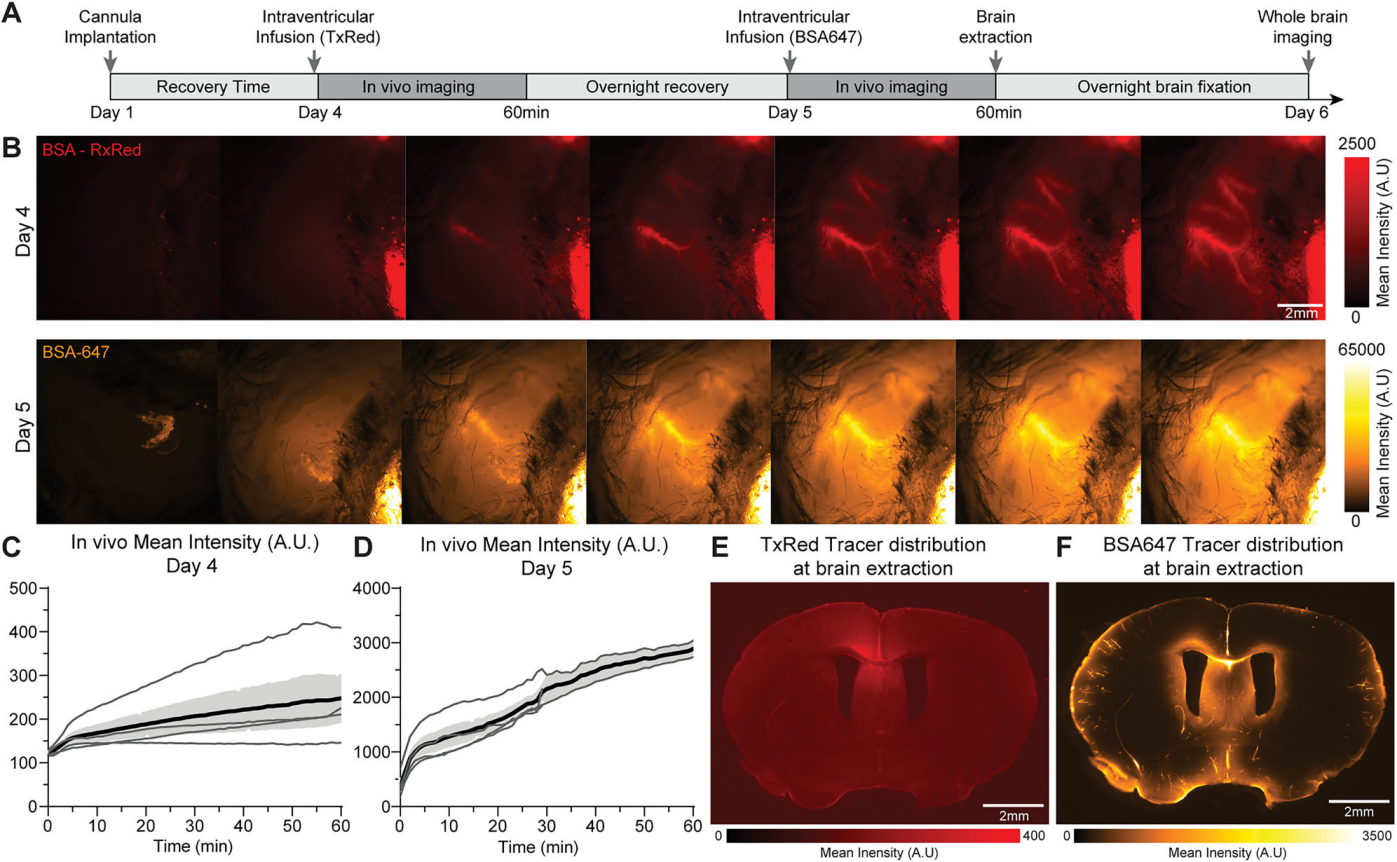
Multiple intraventricular infusions can be administered to the same animal. ***A***, Timeline of the experiment. ***B***, Top, Contralateral in vivo imaging at Day 4 of a mouse with an IVC infusion, BSA-TxRed infused and circulated for 60 min. Bottom, Same mouse, contralateral in vivo imaging of BSA-647 infused and circulated for 60 min. ***C***, Mean intensity (A.U.) of BSA-TxRed tracer increases over time, quantified from regions of interest (ROIs) drawn on the middle cerebral artery (MCA; *n* = 4). ***D***, IVC mean fluorescent intensity (A.U.) of BSA-647 tracer increases over time, quantified from ROIs on the MCA (*n* = 4). ***E***, Representative brain section of BSA-TxRed tracer distribution at brain extraction. ***F***, Representative slice of BSA-647 tracer distribution at brain extraction.

Another advantage of the IVC is its ability to enable repeated delivery of tracers in the same experimental animal over several days. This capability allows for the study of glymphatic modulation in response to treatment or temporal changes, addressing a significant limitation of CMC. To demonstrate feasibility of chronic infusions, cannulas were implanted in the right ventricle of mice, followed by a 72 h recovery period. Tracers were then injected on two consecutive experimental days (Fig. 4*A*).

On the first day, mice were infused with the cerebrospinal fluid tracer TxRed (3 kDa, 2% wt/vol in artificial CSF, 10 µl, infused at 2 µl/min) and tracer circulation was imaged in vivo in the contralateral hemisphere for one hour (Fig. 4). On the second day, the mice were reinfused with BSA-647 (66 kDa, 0.5% wt/vol in artificial CSF, 10 µl, infused at 2 µl/min), and the in vivo imaging session was repeated for another hour.

In vivo macroscopic imaging of the exposed mouse skull (Fig. 4*B*) during both sessions showed that, for both tracers, CSF tracer was transported from the cisterna magna up along the perivascular space surrounding the middle cerebral artery in a similar manner (Fig. 4*C*,*D*). The absence of a signal in the baseline images at the start of the second imaging session confirmed that the circulations of the two tracers were independent from each other. Ex vivo analysis of brain slices further demonstrated that the TxRed tracer had been cleared from the brain, while the BSA-647 tracer exhibited normal glymphatic distribution patterns (Fig. 4*E*,*F*).

### Intraventricular cannulation effects on brain

**Figure 5.**
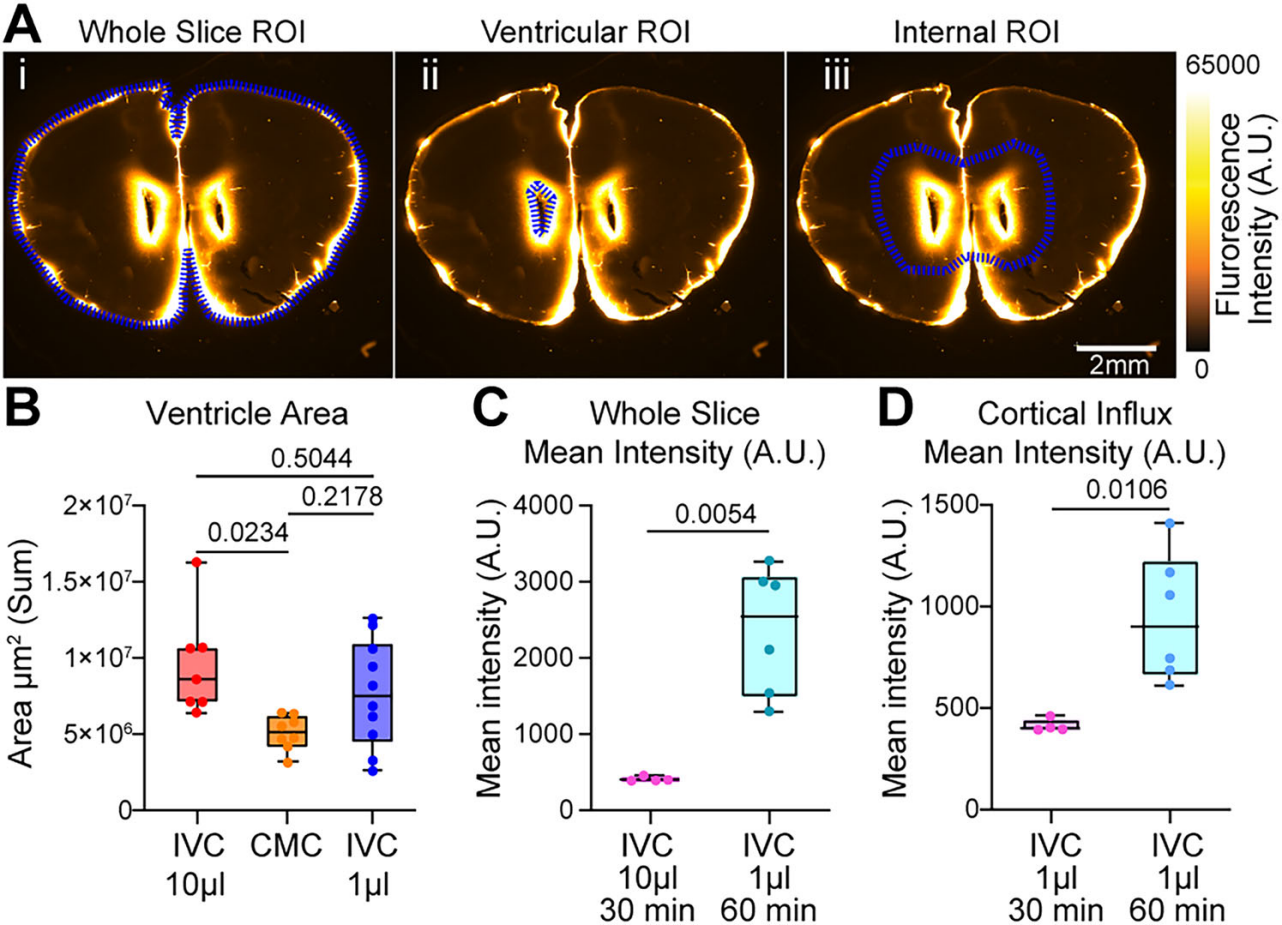
Intraventricular cannula implantation may expand ventricles independent of infusion volume and 1 µl infusion volume labels glymphatic influx. ***A***, Representative brain slices +1.20 mm from bregma after being infused with a solution of BSA-647. ***i***, Representative region of interest (ROI) used to measure whole slice mean fluorescence intensity (A.U.). ***ii***, Representative ROI used to measure ventricle size. ***iii***, Representative internal ROI used to exclude ventricular fluorescence. Scale bar, 2 mm. ***B***, Ventricular area quantified from six brain slices of each mouse with different infusion volumes. IVC 10 µl, 2 µl/min, 30 min of circulation time (*n* = 7). CMC 10 µl, 2 µl/min, 30 min of circulation time (*n* = 8). IVC 1 µl, 0.2 µl/min, 30 min of circulation time (*n* = 10). Statistical difference between IVC 10 µl 30 min and CMC 10 µ 30 min. No statistical difference between CMC 10 µl 30 min and IVC 1 µl. ***C***, Mean intensity (A.U.) of whole brain slices from mice infused with 1 µl BSA-647, 30 min of circulation time versus mice infused with 1 µl, BSA-647, 60 min of circulation time. ***D***, Mean intensity (A.U.) of cortical influx brain slices from mice infused with 1 µl BSA-647, 30 min of circulation time versus mice infused with 1 µl, BSA-647, 60 min of circulation time. Groups were compared using ordinary one-way ANOVA. Statistical comparisons are included in the bar plots.

A common side effect of IVC delivery is a significant localized difference in ventricle size at the site of injection (Cohen-Pfeffer et al., 2017), which we also observed in mice with IVC compared to those with CMC. To determine whether this effect was caused by the infusion process or the presence of the cannula itself, mice with implanted cannulas were infused with varying volumes of the tracer BSA-647(66 kDa, in artificial CSF). Mice received either 10 µl 0.5% wt/vol infused at 2 µl/min rate or 1 µl 1% wt/vol infused at 0.2 µl/min rate. When compared with CMC used as control, the IVC 10 µl group showed a significant difference in ventricular area (*p* = 0.0234), while the IVC 1 µl group did not (*p* = 0.2178). However, no significant difference was observed between the IVC 10 µl and IVC 1 µl groups (*p* = 0.5044; Fig. 5*B*). These findings indicate that the changes in ventricle volume changes are a consequence of the chronic ventricular cannulation itself, rather than the tracer infusion volume.

**Figure 6.**
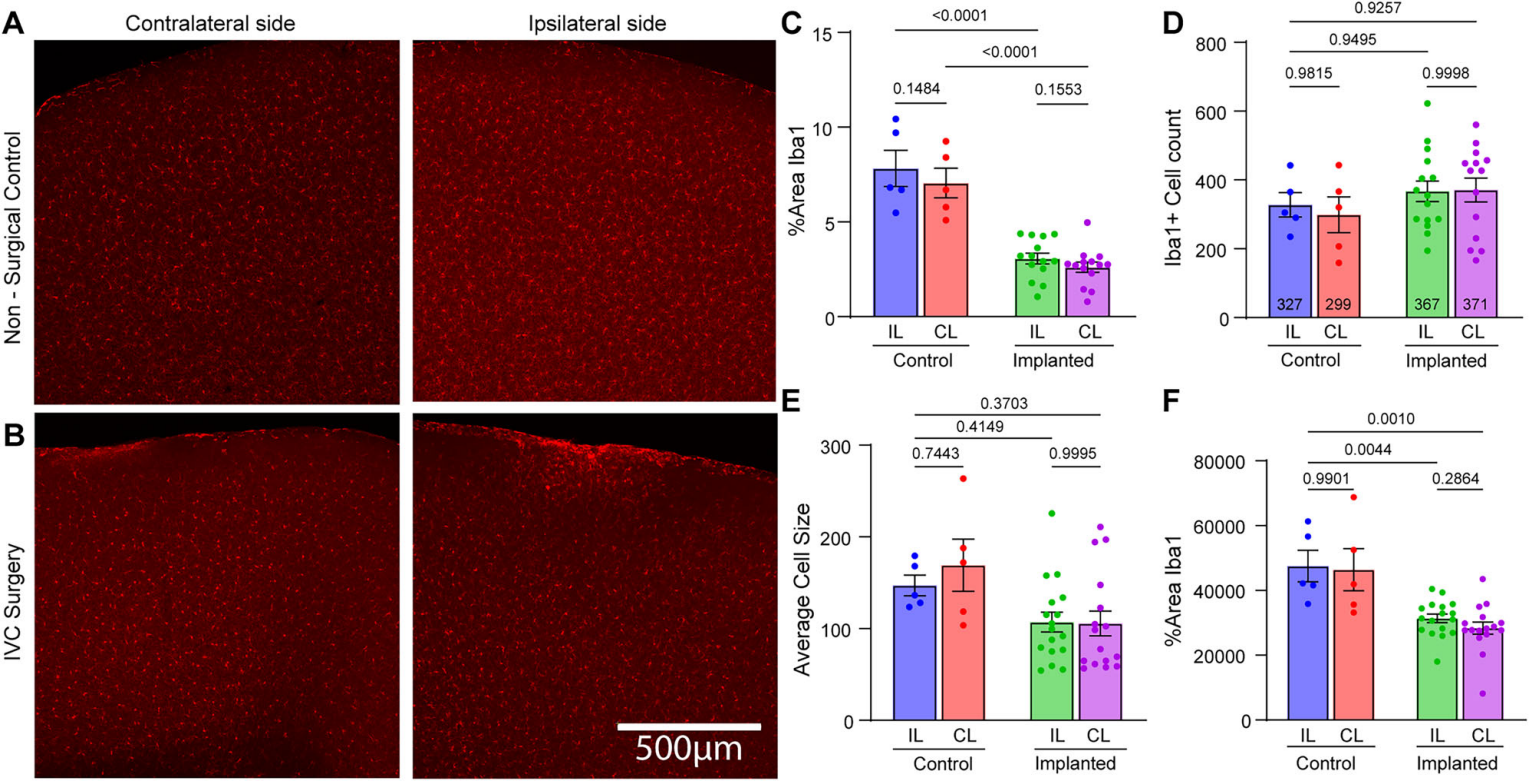
Iba-1 microglial expression increases in IVC. No difference between microglial Iba-1 expression between surgery site and contralateral side. ***A***, Representative contralateral and ipsilateral brain slices 1.80 mm from bregma from nonsurgical control mice after being labeled for Iba-1 through Immunohistochemistry. ***B***, Representative contralateral and ipsilateral brain slices 1.80 mm from bregma from IVC mice after being labeled for Iba-1 through Immunohistochemistry. ***C***, Ipsilateral (IL) and contralateral (CL) percentage area covered by Iba1 in control (*n* = 5) versus cannula implanted mice (*n* = 15). ***D***, Ipsilateral (IL) and contralateral (CL) number of cells labeled for Iba1 between control (*n* = 5) and cannula implanted mice (*n* = 15). Number of cells ipsilateral control = 327, contralateral control = 299, ipsilateral implanted 367, contralateral implanted = 371. ***E***, Ipsilateral (IL) and contralateral (CL) average cell size between control (*n* = 5) and cannula implanted mice (*n* = 15). ***F***, Ipsilateral (IL) and contralateral (CL) area covered (in pixels) by Iba1 in control (*n* = 5) versus cannula implanted mice (*n* = 15). Groups were compared using mixed effects analysis. Statistical comparisons are included in the bar plots. Scale bar, 500 µm.

To rule out the possibility that ventricle enlargement was associated with a neuro-inflammatory response to the cannula implantation, we assessed microglial reactivity using Iba-1 immunostaining. Compared with non-surgical controls, microglia cell density increased locally at the site of the cannula implantation, but no significant differences in Iba-1 expression were observed between the surgery site and the contralateral side of the IVC implanted mice (Fig. 6). Combined, these results demonstrate that changes in ventricle morphology do not alter glymphatic CSF tracer influx at the contralateral hemisphere.

## Discussion

We present a detailed protocol for the successful delivery of fluorescent tracers into the ventricular CSF of mice, enabling the study of glymphatic transport in vivo and in freely behaving mice. Intraventricular infusions have proved to be a great approach for the delivery of drugs to the brain. Intraventricular cannulations have been widely used for different studies on CSF delivery (Delgado et al., 1952; Feldberg and Sherwood, 1953a,b). Previous studies using intraventricular cannulations have proven to be successful for the delivery of viruses (Murlidharan et al., 2016) and nanoprobes (Pessa, 2022) and to be attainable using different cannulas (Kokare et al., 2011). Glymphatic transport has proven to be manipulable using IVC and ultrasounds (Ye et al., 2023). Here, we demonstrate that the well-stablished LV infusion method also can be used as an alternative approach for studying the glymphatic system. Direct infusions to the CSF bypass the blood–brain barrier, improving the control and efficacy of the delivery (Ng et al., 2014; Rishab et al., 2015). Earlier studies on acute intraventricular infusions showed that the procedure disrupted CSF transport and impaired glymphatic function (Iliff et al., 2012; Mestre et al., 2018). However, subsequent studies highlighted the importance of surgery timing, showing that glymphatic flow is restored 24 h or more after surgery (Plá et al., 2022). The data presented here replicated this finding, demonstrating an acute impairment of CSF transport immediately following implantation (Fig. 3*Aii*). Importantly, allowing adequate recovery time for the mice ensures that CSF circulation returns to baseline levels, providing an alternative approach for assessing glymphatic function.

Since the discovery of the glymphatic system in 2012 (Iliff et al., 2012), CMC has been widely used (Iliff et al., 2013; Hablitz et al., 2019, 2020; Giannetto et al., 2020). CMC, whether using acute or chronic cannula implantation in the cisterna magna, has enabled comprehensive characterization of CSF influx patterns, with tracer accumulating in the perivascular space surrounding arteries and at cisterns the base of the brain (Xavier et al., 2018). Yet, chronic implantation has proven problematic, with difficulties in achieving stable fixation due to anatomical limitations and the discomfort experienced by the mice. This discomfort can influence results, given the glymphatic system's sensitivity to norepinephrine levels (Benveniste et al., 2019). Thus, there is a clear need for a procedure that allows for the delivery of CSF tracers into the non-anesthetized mice, enabling tracking and analysis of glymphatic flow under awake and natural sleep conditions.

Our analysis shows that tracers delivered via intraventricular injections, like those delivered by cisterna magna injections, labels the circle of Willis, as well as the pial and intraparenchymal perivascular spaces (Figs. 2, 3). However, intraventricular injections uniquely allow tracers to penetrate periventricular brain tissue, which is not typically observed with cisterna magna infusions. This enables the evaluation of the permeability of the brain–CSF interface. Adjusting the analysis to exclude the ventricular and periventricular tissue signal revealed that cortical tracer distribution after intraventricular injections replicates the patterns seen with cisterna magna injection. Furthermore, transcranial in vivo macroscopic imaging showed that the CSF tracers enter the perivascular space surrounding the middle cerebral artery, enabling real-time quantification of perivascular CSF tracer influx. Additionally, intraventricular cannulations provide chronic access to the ventricles, allowing longitudinal studies. These include reassessing of glymphatic function over time through repeated tracers' infusion at different experimental intervals, as demonstrated here, or IVC administration of therapeutics. IVC delivery treatments bypasses the blood–brain barrier, facilitating the delivery of drugs restricted from entering the brain while reducing the risk of systemic adverse reactions (Lohela et al., 2022; Fahoum and Eyal, 2023). In both cases, this method enables the study of CSF circulation or therapeutic interventions under physiological conditions, despite the presence of a cannula.

However, a potential limitation of intraventricular injections is the associated increase in ventricle volume. To investigate this, we examined the effects of varying the volume and duration of CSF tracer infusions on ventricular expansion and perivascular labeling (Fig. 5*B–D*). These manipulations failed to eliminate ventricular expansion, indicating that the mere presence of the intraventricular cannula is responsible for the enlargement of the ventricles, consistent with previously described non-infectious complications (Cohen-Pfeffer et al., 2017). Despite this, IVC devices have been used for decades in the treatment various pediatric and adult central nervous system disorders (Slavc et al., 2018). Another potential drawback of intraventricular cannulation is the localized inflammation near the implantation site. To analyze microglia activation, we performed immunohistochemistry staining for Iba1 protein. Mice with cannulas implanted for at least 72 h showed signs of microglia activation localized to the implantation site. However, further analysis revealed that the area covered by Iba1 staining, as well as microglial cell size and count, were similar between control mice and IVC mice, at both ipsilateral and contralateral sites. This suggests that cannula implantation affects microglia expression locally at the site but does not induce widespread microglial activation across the brain (Fig. 6).

**Figure 7.**
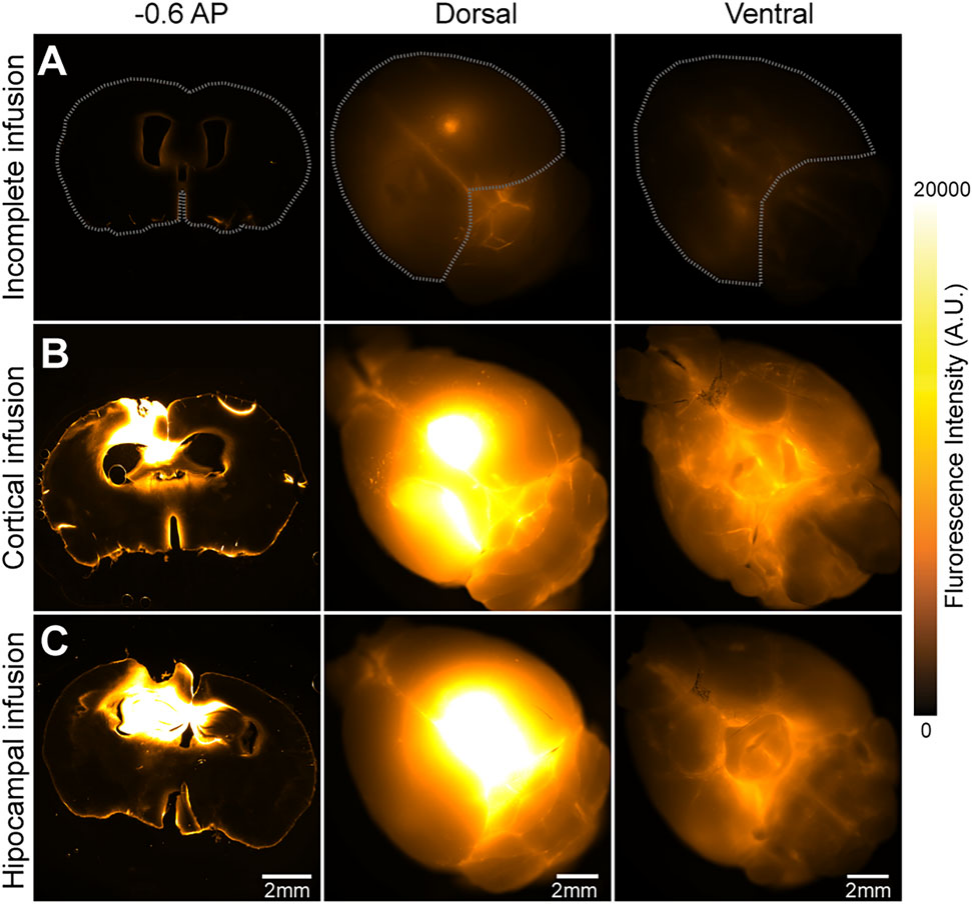
Common surgical mistakes prevent normal tracer influx. ***A***, Representative −1.2 mm from bregma brain slice, dorsal and ventral view of a brain with IVC that did not receive a full infusion of BSA-647. ***B***, Representative −1.2 mm from bregma brain slice, dorsal and ventral view of a brain with IVC in which the cannula did not reach the ventricle. The BSA-647 tracer was instead infused into the brain cortex. ***C***, Representative −1.2 mm from bregma brain slice, dorsal and ventral view of a brain with IVC in which the cannula was inserted in the hippocampus instead of the ventricle. Scale bar, 2 mm.

Lastly, another limitation of intraventricular cannulations is the invasive nature and the high level of precision required to prepare, localize, and implant the cannula inside the ventricle without damaging surrounding brain regions. Minor deviations in surgical coordinates and issues with the cannula line can result in incomplete infusions, cortical infusions, or hippocampal infusions (Fig. 7). Such errors can lead to improper labeling of the brain parenchyma, disrupting glymphatic flow and altering tracer distribution. Despite these challenges, enduring adequate recovery times and proper surgical training can yield results comparable with those achieved by CMC cannulations, while offering a more reliable entry point to the CSF compartment. This method expands the range of experimental possibilities, including studying CSF influx in freely moving mice, analyzing glymphatic flow during the natural sleep/wake cycle, or performing behavioral assessment while infusing mice with various CNS active drugs.

## Synthesis

Reviewing Editor: Matthew Grubb, King's College London Decisions are customarily a result of the Reviewing Editor and the peer reviewers coming together and discussing their recommendations until a consensus is reached. When revisions are invited, a fact-based synthesis statement explaining their decision and outlining what is needed to prepare a revision will be listed below. The following reviewer(s) agreed to reveal their identity: NONE.

N/A
